# Developmental delay and late onset HBSL pathology in hypomorphic *Dars1*^*M256L*^ mice

**DOI:** 10.1007/s11064-022-03582-4

**Published:** 2022-03-31

**Authors:** Matthias Klugmann, Elizabeth Kalotay, Fabien Delerue, Lars M. Ittner, Andre Bongers, Josephine Yu, Margaret J. Morris, Gary D. Housley, Dominik Fröhlich

**Affiliations:** 1 Translational Neuroscience Facility, Department of Physiology, School of Medical Sciences, University of New South Wales, 2052 Sydney, NSW Australia; 2 Dementia Research Centre, Department of Biomedical Sciences, Faculty of Medicine, Health and Human Sciences, Macquarie University, 2109 Sydney, NSW Australia; 3 Biomedical Resources Imaging Laboratory, University of New South Wales, 2052 Sydney, NSW Australia; 4 Department of Pharmacology, School of Medical Sciences, University of New South Wales, 2052 Sydney, NSW Australia

**Keywords:** Hypomyelination with brainstem and spinal cord involvement and Leg spasticity, HBSL, Leukodystrophy, *DARS1*, Aminoacyl-tRNA synthetase, Aspartyl-tRNA synthetase

## Abstract

The leukodystrophy Hypomyelination with Brainstem and Spinal cord involvement and Leg spasticity (HBSL) is caused by recessive mutations of the *DARS1* gene, which encodes the cytoplasmic aspartyl-tRNA synthetase. HBSL is a spectrum disorder with disease onset usually during early childhood and no available treatment options. Patients display regression of previously acquired motor milestones, spasticity, ataxia, seizures, nystagmus, and intellectual disabilities. Gene-function studies in mice revealed that homozygous *Dars1* deletion is embryonically lethal, suggesting that successful modelling of HBSL requires the generation of disease-causing genocopies in mice. In this study, we introduced the pathogenic *DARS1* ^*M256L*^ mutation located on exon nine of the murine *Dars1* locus. Despite causing severe illness in humans, homozygous *Dars1* ^*M256L*^ mice were only mildly affected. To exacerbate HBSL symptoms, we bred *Dars1* ^*M256L*^ mice with *Dars1*-null ‘enhancer’ mice. The *Dars1* ^*M256L/−*^ offspring displayed increased embryonic lethality, severe developmental delay, reduced body weight and size, hydrocephalus, anophthalmia, and vacuolization of the white matter. Remarkably, the *Dars1* ^*M256L/−*^ genotype affected energy metabolism and peripheral organs more profoundly than the nervous system and resulted in reduced body fat, increased respiratory exchange ratio, reduced liver steatosis, and reduced hypocellularity of the bone marrow. In summary, homozygous *Dars1* ^*M256L*^ and compound heterozygous *Dars1* ^*M256L/−*^ mutation genotypes recapitulate some aspects of HBSL and primarily manifest in developmental delay as well as metabolic and peripheral changes. These aspects of the disease might have been overlooked in HBSL patients with severe neurological deficits but could be included in the differential diagnosis of HBSL in the future.

## Introduction

Hypomyelination with Brainstem and Spinal cord involvement and Leg spasticity (HBSL) is caused by recessive missense mutations of the *DARS1* gene encoding the cytoplasmic aspartyl-tRNA synthetase (AspRS) [1]. HBSL belongs to the diverse group of leukodystrophies – neurogenetic diseases of the brain white matter characterized by an early onset, severe course, substantial mortality, and lack of treatment options. The population incidence of leukodystrophies is about one in 7,600 [2].

AspRS is one of the 36 human cytosolic and mitochondrial aminoacyl-tRNA synthetases (AaRSs), which play a pivotal role in translation by charging tRNAs with cognate amino acids thus ensuring the fidelity of protein synthesis [3]. AaRS deficiencies are not tolerated and primarily manifest in severe neurological diseases including encephalopathies, neuropathies and leukodystrophies indicating increased susceptibility of neural cells to disturbed protein synthesis [4]. The first mutations in AaRS-encoding genes were identified almost two decades ago as the cause of the neuropathy Charcot-Marie-Tooth disease [5]. To date, pathological mutations that result in neurological diseases have been identified in 24 AaRS genes [6].

HBSL is a spectrum disorder with disease onset usually around 3–36 months. Patients display regression of previously acquired motor milestones, spasticity, ataxia, seizures, nystagmus, and intellectual disabilities [1, 3]. The HBSL index patient passed away at the age of ten. Magnetic resonance imaging (MRI) of patients with infantile disease onset shows signal abnormalities of the supratentorial and spinal cord white matter indicative of hypomyelination. A similar disease presentation was observed for the leukodystrophy Leukoencephalopathy with Brainstem and Spinal cord involvement and elevated Lactate (LBSL), which is caused by mutations of the mitochondrial homolog *DARS2* [7]. HBSL and LBSL affect the same central nervous system (CNS) structures and their unique MRI pattern sets them apart from other leukodystrophies [1]. Recently, HBSL patients with late disease onset, milder relapsing-remitting course, responsiveness to steroids, and focal white matter changes suggestive of an inflammatory demyelinating disorder such as multiple sclerosis have been reported (Wolf et al. 2015). Therefore, many HBSL patients might remain undiagnosed and HBSL prevalence could be higher than originally anticipated. In HBSL, genotype to phenotype correlations have been challenging to establish in the absence of accurate disease models and these models are urgently needed for the development of effective treatment options. In previous studies, we have established two transgenic HBSL models. Homozygous *Dars1* knockout is embryonically lethal [8], while mice carrying a hypomorphic *Dars1* ^*D367Y*^ allele *in trans* to a deletion allele present with early developmental delay, hydrocephalus, hypomyelination and white matter vacuolization, followed by late onset motor impairment [9]. Moreover, we have identified that AspRS expression in the brain of both mice [8] and humans [10] is enriched in neuronal lineage cells, with far lower expression in glia.

In this study we introduced the human HBSL-causing *DARS1* ^*M256L*^ mutation into the mouse *Dars1* gene using CRISPR/Cas9 gene editing. This mutation is located within the catalytic domain of AspRS and is expected to lower enzymatic activity. Four of the ten initially described HBSL cases were homozygous for this mutation [1]. Despite resulting in severe illness in humans, mice carrying the *Dars1* ^*M256L*^ mutation homozygously were only mildly affected. To trigger HBSL symptoms in *Dars1* ^*M256L*^ mice, we bred them with *Dars1*-null ‘enhancer’ mice as previously achieved for the *Dars1* ^*D367Y*^ mutation [9]. Homozygous *Dars1* ^*M256L*^ and compound heterozygous *Dars1* ^*M256L/−*^ mutation genotypes only partially recapitulated the pathological spectrum of HBSL in mice, and resulted in developmental delay, as well as peripheral and metabolic changes – aspects of the disease that have not previously been noted in HBSL patients. Taken together, the *Dars1* ^*M256L/−*^ transgenics described here will be a valuable model to explore novel diagnostic tools and experimental therapies; and will inform on peripheral abnormalities contributing to the complex HBSL pathophysiology.

## Methods

### Ethics

All procedures were approved by the University of New South Wales Animal Care and Ethics Committee and were conducted in accordance with the Australian Code of Practice for the Care and Use of Animals for Scientific Purposes.

### Animals

Mice were group housed in ventilated cages and fed *ad libitum* with standard chow diet. The nineth exon of the murine *Dars1* gene (ENSEMBL ENSMUSG00000026356) was targeted to introduce the c.766A > C p.Met256Leu mutation by CRISPR-mediated gene targeting. A single guide RNA (sgRNA 5’-CACATCTGCTTATACAATTG-3’) was rationally designed using a computational tool to minimize off-targets (http://crispr.mit.edu) and was produced using a non-cloning method whereby a T7-conjugated forward primer generates a linear template by PCR, as previously described [11]. Briefly, the sgRNA scaffold of the pX330 (Addgene; #42,230; gift from Dr. Feng Zhang) was used to synthesize a small linear DNA template. Next, this template was *in-vitro* transcribed using a T7 Quick High Yield RNA synthesis kit (NEB; #E2050S) following the manufacturer’s instructions. The resulting sgRNA was purified using NucAway Spin columns (ThermoFisher; #AM10070) and incubated with S.p.Cas9 protein (NEB; #M0646T) to form ribonucleoprotein complexes (RNPs). RNPs were mixed with a single-stranded oligonucleotide (5’-ACTGTGTCCTATTTTAAAAATAATGCCTACCTTGCTCAGTCTCCACAATTGTATAAGCAGCTGTGCATTTGTGCTGATTTTGAGAAGGTTTTCTGCATTGGACCAGGTAAGATATTGTCA-3’) and electroporated (NEPA21, Nepagene) into fertilised C57Bl/6J zygotes with the respective concentrations: 100 ng/µl Cas9, 200 ng/µl sgRNA and 300 ng/µl oligo. Five live pups were produced, two of which carried the expected mutation.

Correct targeting was validated by Sanger sequencing. An 849 bp fragment containing the c.766 A > C mutation was amplified by PCR using the following primers: 5′- AGCTCACTTTGTAGGCTGGC-3′ (forward) and 5′- GCCACATCCCCTGCTCTTAA-3′ (reverse). The amplicon was purified by ethanol precipitation and sequenced using the forward primer.

In order to generate compound heterozygous *Dars1* ^*M256L/−*^ mice, homozygous *Dars1* ^*M256L*^ mice were bred with heterozygous *Dars1*-null carriers [8]. Genotyping of the *Dars1*-null allele was performed as described [8].

### Behavioral testing

Behavioral tests were conducted in homozygous *Dars1* ^*M256L*^ mice with age- and sex-matched wildtype mice as controls. Behavior of *Dars1* ^*M256L/−*^ mice was compared to *Dars1* ^*M256L/+*^ littermates. All behavioral tests were conducted in the afternoon to ensure comparability across groups. Muscle strength and motor coordination were assessed using the hanging wire test as described previously [12]. Locomotor behavior was assessed using a rotarod apparatus (Ugo Basile, Italy) as previously outlined [8]. Mice were tested in three trials per day on two consecutive days (six trials in total) and the latency to fall was averaged over six trials.

The open field-test was performed in an open box (40 × 40 × 40 cm^3^) under bright light conditions (100 lx) as described previously [9]. Total distance travelled, distance travelled in the inner compartment, and time spent in the inner compartment during a 30 min trial were analyzed using ANY-Maze™ tracking software (Stoelting, USA).

The acoustic startle response (ASR) and the pre-pulse inhibition (PPI) were measured using the SR-LAB Startle Response System (San Diego Instruments, USA) as previously detailed [8, 13]. The ASR was determined in response to 40 ms sound stimuli with increasing intensities ranging from 60 to 120 dB sound pressure level (SPL). The PPI was determined as the decrease of the ASR amplitude following a 120 dB SPL startle pulse preceded by three different pre-pulses (72, 76, or 80 dB SPL).

### Indirect Calorimetry

Respirometry analyses were performed by indirect calorimetry using the Comprehensive Laboratory Animal Monitoring System (CLAMS; Columbus Instruments, USA) as previously described [14]. Mice were maintained in CLAMS cages for 24 h acclimatization followed by a 24 h recording period, allowing simultaneous measurement of metabolic parameters including oxygen consumption, carbon dioxide production, respiratory exchange ratio (RER), food intake and activity levels. Measurements of 1.5 min were sampled every 15 min throughout the recording period.

### Magnetic resonance imaging (MRI) and body composition analysis

MRI was performed using a 9.4T BioSpec Avance III 94/20 (Bruker, Germany) magnetic resonance microimaging system equipped with a 15 mm internal diameter quadrature specimen volume coil for radiofrequency transmission and reception as described [9]. Body composition analysis was performed using the EchoMRI-900™ system equipped with an A100 mouse antenna insert (EchoMRI LLC, USA) as outlined before [12]. Fat and lean mass were calculated as proportion of total body mass.

### Mouse histopathology

This study utilized the Phenomics Australia (PA) Histopathology and Slide Scanning Service at the University of Melbourne, Australia. This service included full necropsy of *Dars1* ^*M256L/−*^ mice in comparison to *Dars1* ^*M256L/+*^ littermates (n = 5 per group). Services performed were as follows: harvesting of 25 organs, fixation, embedding in paraffin, sectioning, staining, slide scanning, and detailed histopathology report. Staining methods employed were Haematoxylin and Eosin (H&E; Fig. 4C and Fig. 6D-G) and Masson’s trichrome (MT; Fig. 6H).

### Immunohistochemistry

Following a lethal injection of pentobarbital, mice were transcardially perfused with 10 ml phosphate buffered saline (PBS) followed by 10 ml 4% paraformaldehyde (PFA). Brains were post-fixed in 4% PFA for 2 h at room temperature and cryoprotected in 30% sucrose. Brains were sectioned coronally (thickness: 40 μm) using a cryostat as described [15]. Following permeabilization in 0.2% TritonX-100 in PBS, sections were stained with FluoroMyelin™ Red Fluorescent Myelin Stain (Thermo Fisher Scientific; #F34652) according to the manufacturer’s instructions. Sections were mounted in Mowiol (Calbiochem, Germany) and imaged using an LSM710 confocal microscope (Zeiss, Germany).

### RNA isolation and qPCR

11-month-old mice were euthanized by cervical dislocation and the brains extracted. The brain regions cortex (CX), cerebellum (CB), brainstem (BS), and basal ganglia (BG) were dissected and snap frozen in liquid nitrogen, followed by homogenization of the brain tissue in liquid nitrogen using mortar and pestle as previously described [10]. RNA was extracted using the RNeasy MiniKit (Qiagen; #74,106) with on-column DNase digest (Qiagen; #79,254). Reverse transcription was performed with the High-Capacity cDNA Reverse Transcription Kit (Applied Biosystems; #4,368,813). Quantitative real-time PCR (qPCR) was performed on a StepOnePlus™ Real-Time PCR system (Applied Biosystems, USA) employing the following TaqMan probes (Applied Biosystems, USA): *Dars1* (Mm00624185_m1), *Plp* (Mm00456892_m1), *Mbp* (Mm01266402_m1), *Cnp* (Mm01306640), *Aspa* (Mm004808667_m1), and *GusB* (Mm01197698_m1). Comparative ΔΔCT values were determined relative to the housekeeper *GusB* and the CX region.

### SDS-PAGE and Western-blotting

Brain tissue from the CX, CB, BS, and BG regions of 11-months-old mice was extracted and homogenized as described above. Brain homogenates were lysed in 10 µl/mg lysis buffer (50 mM Tris-Cl, pH 7.4, 1 mM EDTA pH 8.0, 250 mM NaCl, and 1% Triton-X) including Roche cOmplete™ protease inhibitor cocktail (Roche, Switzerland). Lysates were sonicated with a Branson 450 Digital Probe Sonifier (10% amplitude) and centrifuged at 10,000 x g in a conventional benchtop centrifuge. Supernatant was transferred to a new tube and protein concentration was measured using the Bradford protein assay (Bio-Rad; #5,000,006).

SDS-PAGE on a 10% acrylamide gel and Western-blotting onto a PVDF membrane (Bio-Rad; #162–0177) was performed as previously described [10]. Following blocking with 4% skim milk in PBS 0.1% Tween (PBS-T) to prevent unspecific binding of antibodies, membranes were probed with primary antibodies: mouse anti-AspRS (1:1000; SantaCruz; #sc-393,275), rat anti-PLP (clone aa3; 1:200, donation from Prof. J. Trotter, Mainz, Germany), mouse anti-CNP (1:3000, Abcam; #ab6319), and rabbit anti-GAPDH (1:4000, Cell Signaling, #2118S). After washing three times for 15 min in PBS-T, membranes were probed with HRP-conjugated secondary antibodies (1:10,000; Dianova, Germany). Membranes were developed with 1 ml Clarity Western ECL substrate (Bio-Rad; #170–5060) and imaged using the ChemiDoc MP system (Bio-Rad, USA).

### Statistics

Graphs and statistical analysis were performed with Prism 8 software (GraphPad, USA). Following validation of normal distribution of data, either a two-way analysis of variance (ANOVA) with Bonferroni’s multiple comparisons post-hoc test or a Student’s t-test was performed as indicated. Values are displayed as mean ± SEM with statistical significance being defined as p < 0.05.

## Results

### Homozygous Dars1^M256L^ mice are only mildly affected

**Fig. 1:**
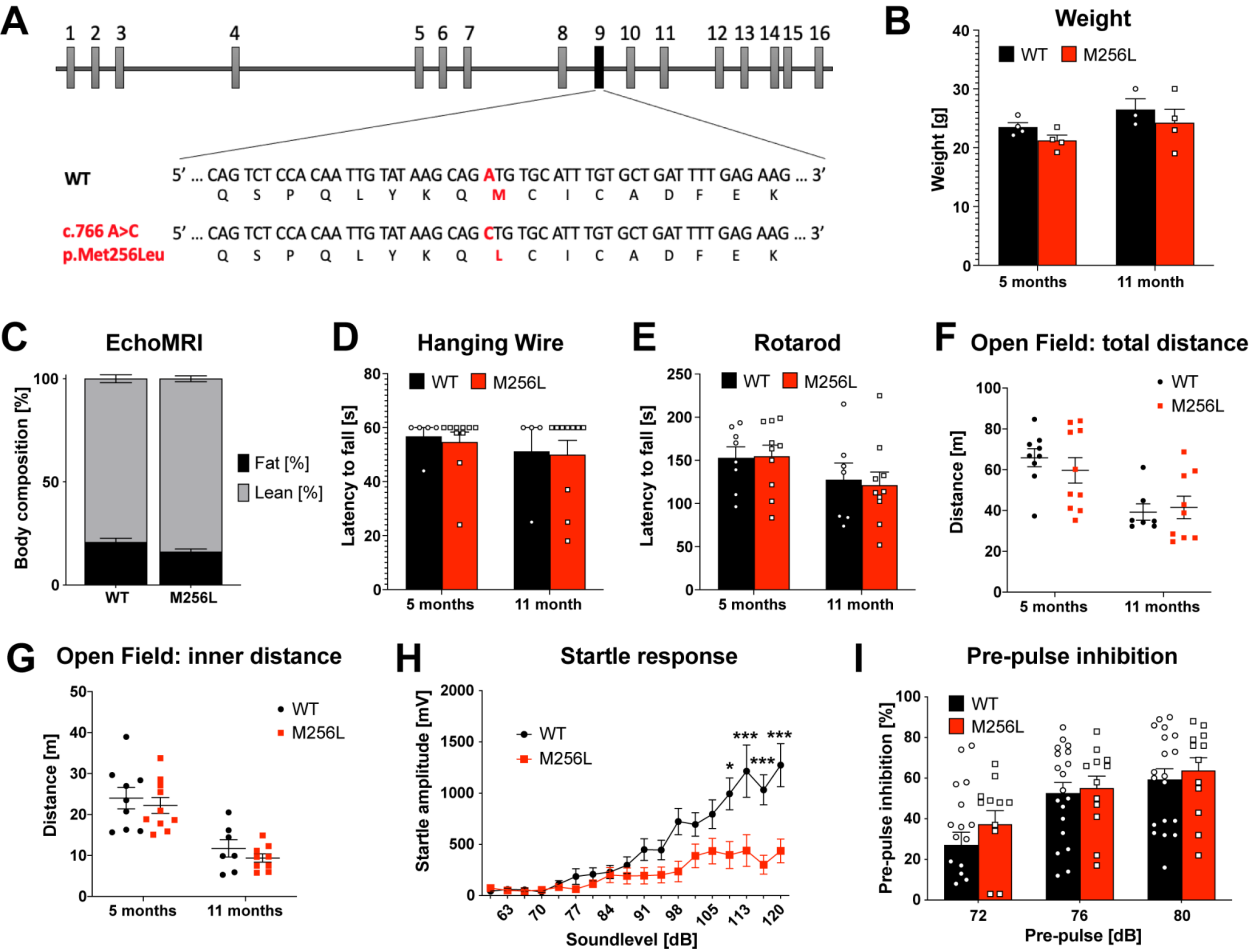
Phenotype of homozygous *Dars1* ^*M256L/M256L*^ mice (M256L). (A) Illustration of the genomic location of the *Dars1* c.766 A > C; p.Met256Leu mutation on exon 9. (B) Body weight of M256L female mice compared to WT controls at 5 and 11 months of age (n = 3–4). (C) Body composition analysis using EchoMRI showed no significant differences between homozygous M256L and WT mice (n = 7–10). (D and E) The hanging wire (D) and the rotarod test (E) revealed normal muscle strength and motor coordination of M256L mice compared to WT controls (n = 4–10). (F and G) Total distance (F) and distance travelled in the inner compartment (G) of an open field test apparatus were comparable between M256L and WT mice (n = 7–10). (H) Five-month-old homozygous M256L mice exposed to 40 ms sound stimuli with increasing intensities (60–120 dB SPL) displayed reduced acoustic startle response compared to controls (n = 10–19). (I) No differences in pre-pulse inhibition (120 dB SPL startle pulse preceded by 72, 76, or 80 dB SPL pre-pulses) were observed in M256L mice compared to WT controls (n = 12–19). Data represent mean ± SEM (*p < 0.05, ***p < 0.001; Two-way ANOVA)

HBSL is caused by recessive point mutations of the *DARS1* gene [1]. Our previous studies revealed that homozygous *Dars1*-null mutation is embryonically lethal in mice [8] and that the hypomorphic *Dars1* ^*D367Y*^ mutation in combination with the *Dars1*-null mutation leads to developmental delay, hydrocephalus, hypomyelination, white matter vacuolization and late onset motor deficits [9]. Here, we introduced the HBSL point mutation c.766 A > C p.Met256Leu (M256L) into the *Dars1* gene using CRISPR/Cas9 gene editing. The single nucleotide change in exon nine alters the wildtype ATG codon to CTG, which translates into leucine instead of methionine (Fig. 1 A). Surprisingly, despite resulting in severe illness in human patients, homozygous *Dars1* ^*M256L*^ mice did not exhibit overt HBSL pathology. Body weight of *Dars1* ^*M256L*^ mice compared to age and sex matched wildtype (WT) controls was unchanged (Fig. 1B). Body composition analysis using EchoMRI indicated a slight yet not significant reduction in fat mass of *Dars1* ^*M256L*^ mice compared to WT controls (Fig. 1 C). Muscle strength and locomotor behavior was assessed using the hanging wire and rotarod tests but revealed no differences between genotypes (Fig. 1D and E). In order to detect early and late onset motor deficits, behavioral tests were conducted at 5 and 11 months, respectively. To identify potential differences in locomotion and explorative behavior, the open field test was performed. *Dars1* ^*M256L*^ mice travelled the same distance overall (Fig. 1 F) as well as in the inner compartment of the apparatus (Fig. 1G) indicating unchanged locomotor activity and explorative behavior. Next, we measured the acoustic startle response (ASR), a reflex following loud acoustic stimuli that can inform on less obvious neurological deficits. Five-month-old homozygous *Dars1* ^*M256L*^ mice exposed to 40 ms sound stimuli with increasing intensities ranging from 60 to 120 dB SPL displayed significantly reduced ASRs compared to WT controls, which could indicate reduced information processing speeds in *Dars1* ^*M256L*^ mice (Fig. 1 H). Pre-pulse inhibition (PPI), a measurement for sensorimotor gating mechanisms [16] was unchanged in *Dars1* ^*M256L*^ mice compared to WT controls (Fig. 1I).

### Developmental deficits of compound heterozygous Dars1^M256L/-^ mice

**Fig. 2:**
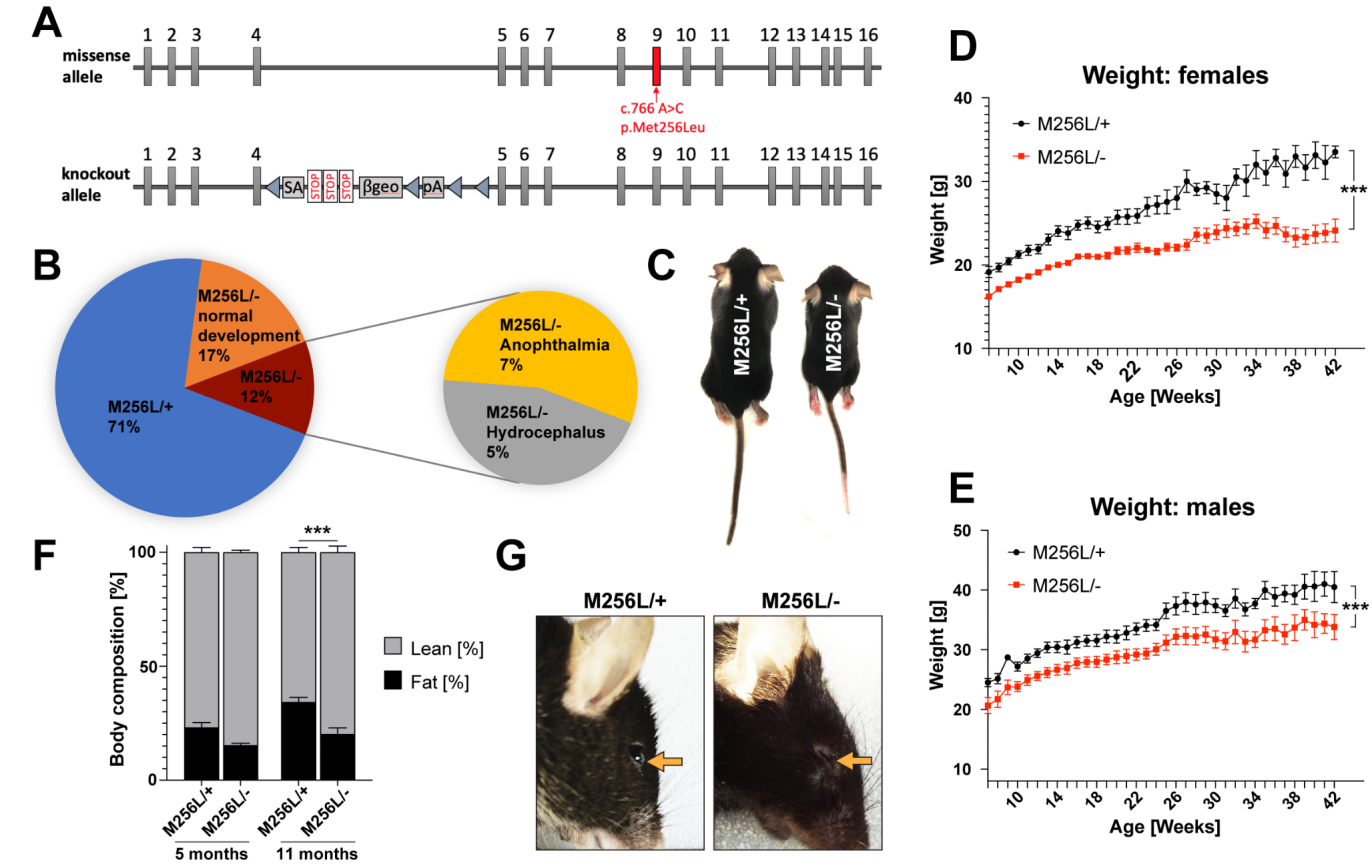
Developmental deficits of compound heterozygous *Dars1* ^*M256L/−*^ mice (M256L/-). (A) Schematic depicting the set of *Dars1* alleles present in compound heterozygous M256L/- mice. Allele 1 contains the c.766 A > C; p.Met256Leu missense mutation while allele 2 is the *Dars1*-null allele described in Fröhlich et al., 2017. (B) Genotype and phenotype distribution in the F1 offspring of homozygous M256L and heterozygous *Dars1*-null mice (n = 94; 19 litters). (C) M256L/- mice show reduced body size compared to age- and sex-matched M256L/+ littermates. (D and E) Weight of M256L/+ and M256L/- females (D; n = 5–11) and males (E; n = 3–11). (F) EchoMRI body composition shows a shift from fat to lean mass in M256L/- mice at 5 months with a significant reduction in body fat in M256L/- mice compared to M256L/+ littermates at 11 months (n = 7–9). (G) 22% of the viable M256L/- mice (7% of the total F1 mice) showed various degrees of anophthalmia or microphthalmia (arrow). Data represent mean ± SEM (***p < 0.001; Two-way ANOVA)

To trigger HBSL symptoms in *Dars1* ^*M256L*^ mice, we bred them with heterozygous *Dars1*-null carriers as previously performed with the *Dars1* ^*D367Y*^ strain [9]. 50% of the offspring were expected to be compound heterozygous for the *Dars1* ^*M256L*^ and *Dars1*-null alleles (Fig. 2A). However, only 29% of the F1 generation (n = 94) had the genotype *Dars1* ^*M256L/−*^ (Fig. 2B). Of the viable *Dars1* ^*M256L/−*^ offspring, 19% (5% of all F1 mice) developed hydrocephalus during the first month of their life and had to be euthanized (Fig. 2B). Additionally, 22% of the *Dars1* ^*M256L/−*^ mice (7% of all F1 mice) were afflicted by microphthalmia or anophthalmia, where one or both eyes are either underdeveloped or completely absent (Fig. 2B and G). In the remaining 59% of the *Dars1* ^*M256L/−*^ offspring (17% of all F1 mice), development was significantly delayed resulting in reduced body size (Fig. 2 C) and weight (Fig. 2D and E). In both, female and male *Dars1* ^*M256L/−*^ mice, weight remained lower throughout life with the difference being more pronounced in female mice (Fig. 2D and E). Body composition analysis employing EchoMRI revealed a shift from fat to lean mass in *Dars1* ^*M256L/−*^ mice at 5 months and a significant reduction in body fat at 11 months (Fig. 2 F) indicating altered energy metabolism in *Dars1* ^*M256L/−*^ mice compared to *Dars1* ^*M256L/+*^ littermates.

### Behavioral assessment of Dars1^M256L/-^ mice

**Fig. 3:**
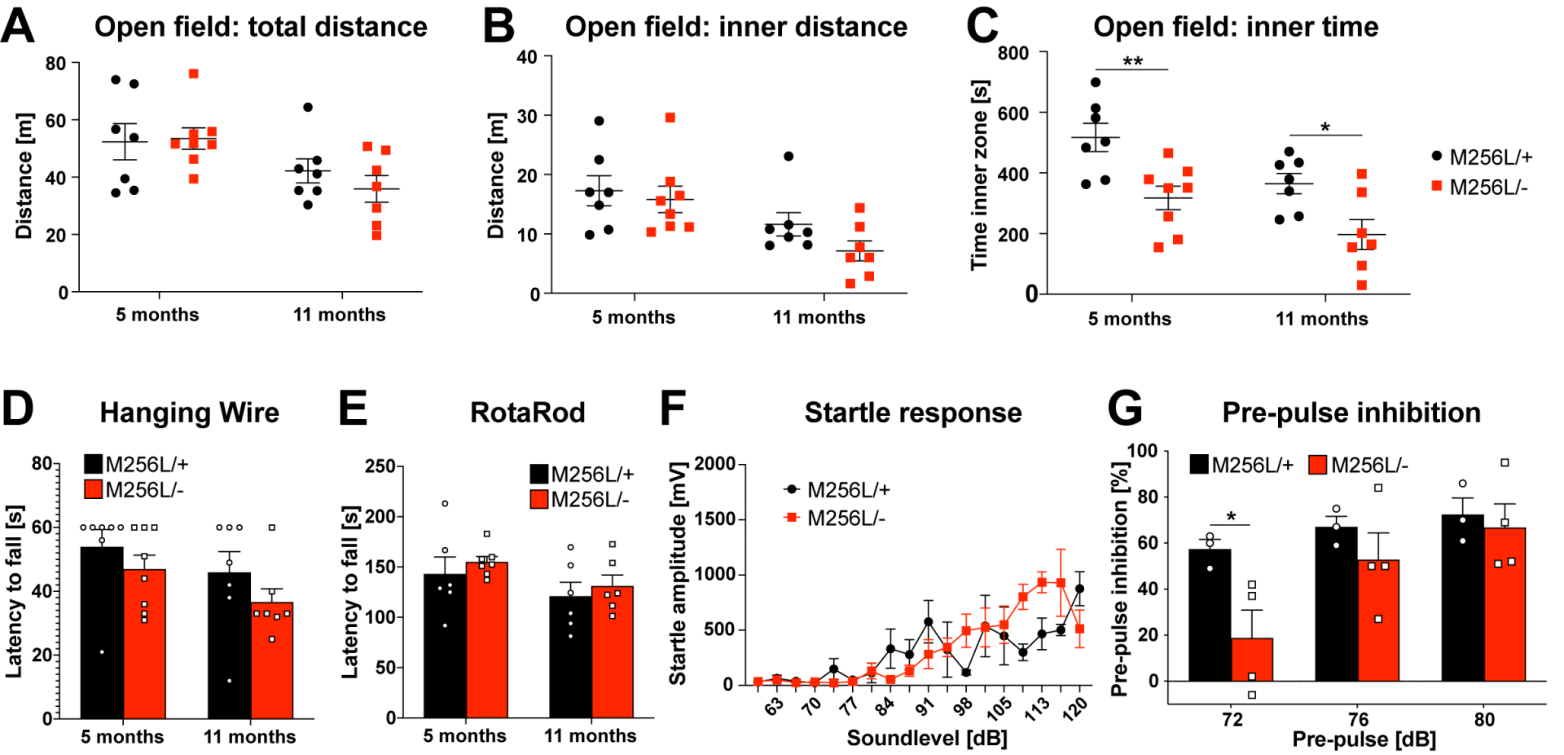
Behavioral assessment of compound heterozygous *Dars1* ^*M256L/−*^ mice (M256L/-). (A-C) Total distance, distance in the inner compartment and time spent in the inner compartment of an open field test apparatus were assessed (n = 7). M256L/- mice spent significantly less time in the inner (open) compartment compared to M256L/+ controls (C). (D and E) Muscle strength and motor coordination assessed by the hanging wire and rotarod tests were unaffected in M256L/- mice compared to M256L/+ controls (n = 7). (F) Acoustic startle responses following sound stimuli with increasing intensities (60–120 dB SPL, 40 ms) were unaltered between M256L/– and M256L/+ mice (n = 4). (G) Pre-pulse inhibition (PPI) through 72, 76 or 80 dB SPL pre-pulses played 100 ms before the 120 dB SPL startle pulse was measured. A significant reduction of PPI was observed in M256L/- mice following the 72 dB SPL pre-pulse (n = 3–4). Data represent mean ± SEM (*p < 0.05, **p < 0.01; Two-way ANOVA)

HBSL patients display severe motor deficits including regression of previously acquired motor milestones, spasticity, ataxia, and seizures [3]. *Dars1* ^*M256L/−*^ mice that overcame the initial developmental deficit and embryonic lethality did not exhibit overt behavioral deficits characteristic of the disease presentation in HBSL patients. We performed a set of motor tests with 5- and 11-month-old *Dars1* ^*M256L/−*^ mice to discriminate early and late onset deficits. Total distance and distance travelled in the inner compartment of an open field test apparatus were unchanged between *Dars1* ^*M256L/−*^ and *Dars1* ^*M256L/+*^ mice demonstrating no differences in locomotor activity and explorative behavior (Fig. 3A and B). Despite travelling the same distance in the inner compartment of the test apparatus, 5- and 11-month-old *Dars1* ^*M256L/−*^ mice spent significantly less time in the inner compartment compared to *Dars1* ^*M256L/+*^ controls (Fig. 3C). The time spent in the inner compartment is thought to reflect anxiety with a reduction indicating increased levels of anxiety. Muscle strength and motor coordination assessed by the hanging wire and rotarod tests were unaffected in *Dars1* ^*M256L/−*^ mice compared to *Dars1* ^*M256L/+*^ controls (Fig. 3D and E). Despite the ASR reduction of homozygous *Dars1* ^*M256L*^ mice compared to WT controls (Fig. 1H), we did not observe a reduction in the ASR of *Dars1* ^*M256L/−*^ mice (Fig. 3F). Conversely, the PPI was significantly reduced in *Dars1* ^*M256L/−*^ mice when a 72 dB SPL pre-pulse preceded the 120 dB SPL startle pulse, with the same trend yet no statistical significance being observed for the 76 and 80 dB SPL pre-pulses (Fig. 3G). Similar results were detected in *Dars1* ^*D367Y*^ mice, where homozygous *Dars1* ^*D367Y*^ mice showed reduced ASRs and compound heterozygous *Dars1* ^*D367Y/−*^ mice presented with impaired PPI [9].

**Fig. 4:**
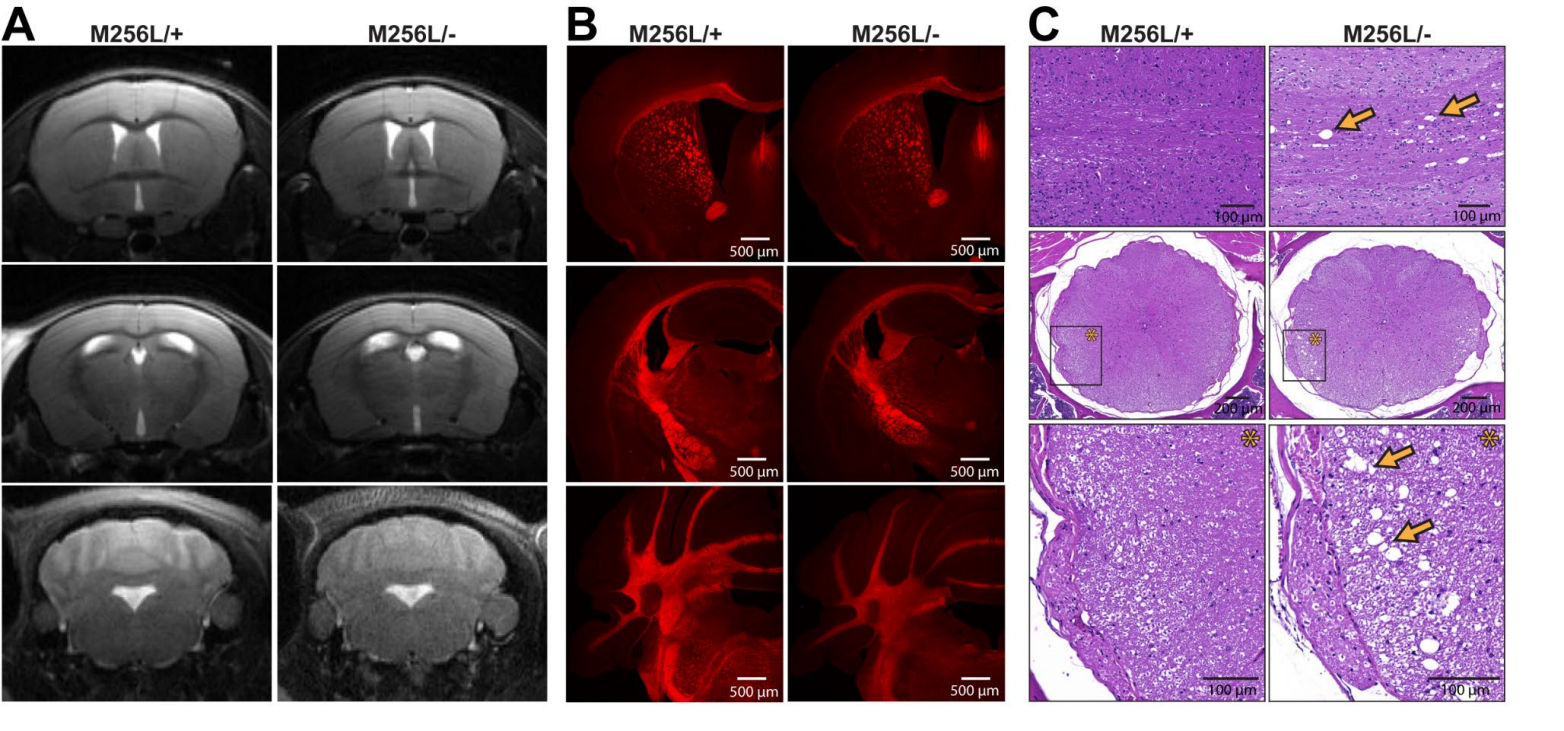
CNS morphology and myelination of compound heterozygous *Dars1* ^*M256L/−*^ mice (M256L/-). (A) Brain MRI did not show overt morphological or myelination abnormalities of M256L/- mice compared to M256L/+ littermates. (B) FluoroMyelin Red staining of coronal brain sections revealed no differences in myelination of M256L/- and M256L/+ mice. (C) Hematoxylin and Eosin (H&E) staining of longitudinal sections (top) and thoracic cross sections (middle and bottom) of the spinal cord showed vacuolization of the lateral white matter (arrows) in 11-month-old M256L/- mice but not in M256L/+ controls

### Dars1^M256L/-^ mice show vacuolization of the spinal cord white matter

MRI of the CNS in HBSL patients shows a distinct pattern of hypomyelination characterized by focal T2 hyperintensities in specific brain and spinal cord regions [1, 17]. To investigate whether *Dars1* ^*M256L/−*^ mice replicate this distinct phenotype, they were subjected to MRI using the Bruker 9.4T BioSpec Avance III 94/20 small animal scanner. Unlike human HBSL patients, *Dars1* ^*M256L/−*^ mice did not show any morphological or myelination abnormalities on brain MR images (Fig. 4 A). To further assess myelination in the brain of *Dars1* ^*M256L/−*^ mice, we performed FluoroMyelin Red stainings on coronal brain sections (Fig. 4B). No myelination differences were observed between genotypes. In the spinal cord, however, H&E staining of longitudinal and thoracic cross sections revealed vacuolization of the lateral white matter in 11-month-old *Dars1* ^*M256L/−*^ mice but not in *Dars1* ^*M256L/+*^ controls (Fig. 4 C). These findings match observations made in *Dars1* ^*D367Y/−*^ mice, which also presented with vacuolization of the lateral and ventral white matter of the spinal cord [9].

### Expression levels of myelin proteins

**Fig. 5:**
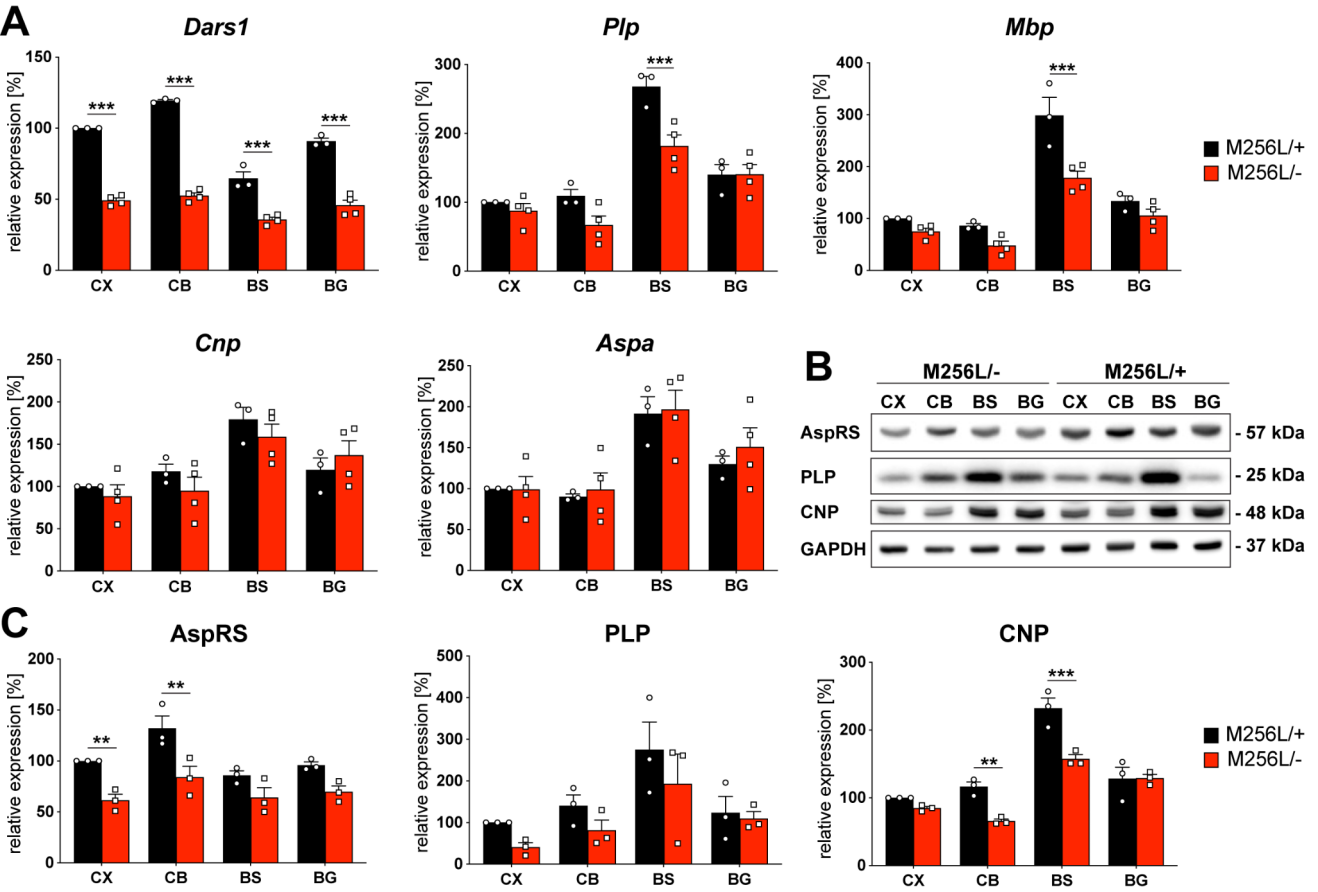
mRNA and protein levels of *Dars1*/AspRS and the major myelin proteins in *Dars1* ^*M256L/−*^ mice (M256L/-). (A) mRNA expression levels assessed by qPCR in different brain regions (CX, cortex; CB, cerebellum; BS, brainstem; BG, basal ganglia) of 11-months old M256L/- mice compared to M256L/+ controls. Expression was normalized to the housekeeper *Gusb* (n = 3–4). (B) Representative Western-blot indicating expression of AspRS, the myelin proteins PLP and CNP, and the housekeeping protein GAPDH in the brain of M256L/- and M256L/+ mice. (C) Densitometric quantification of AspRS, PLP and CNP protein levels normalized to the housekeeper GAPDH in different brain regions of 11-month-old M256L/- mice compared to M256L/+ controls (n = 3). Data represent mean ± SEM (**p < 0.01, ***p < 0.001; Two-way ANOVA)

In addition to white matter vacuolization, *Dars1* ^*D367Y/−*^ mice showed a significant reduction of the major myelin proteins proteolipid protein (PLP), 2’,3’-cyclic nucleotide 3’ phosphodiesterase (CNP) and myelin basic protein (MBP) in the hindbrain of 10-month-old mice [9]. To determine whether a similar effect could be observed in *Dars1* ^*M256L/−*^ mice, we analyzed brain tissue of 11-month-old *Dars1* ^*M256L/−*^ mice and compared it to *Dars1* ^*M256L/+*^ controls. We included analysis of *Dars1* mRNA and AspRS protein to confirm reduction in the cortex (CX), cerebellum (CB), brainstem (BS) and basal ganglia (BG) of *Dars1* ^*M256L/−*^ mice. Expectedly, *Dars1* mRNA was reduced by 50% compared to *Dars1* ^*M256L/+*^ controls in all four brain regions (Fig. 5A). AspRS protein levels were lowered by 50% in the CX and CB of *Dars1* ^*M256L/−*^ mice, however, AspRS protein reduction did not reach statistical significance in the BS and BG (Fig. 5B and C). The myelin protein mRNAs *Plp* and *Mbp* were significantly reduced in the BS of *Dars1* ^*M256L/−*^ mice, while *Cnp* and the oligodendroglial marker *Aspa* mRNA levels remained unchanged (Fig. 5 A). At the protein level, only CNP expression was significantly lowered in the CB and BS of *Dars1* ^*M256L/−*^ mice (Fig. 5B and C). Taken together, there is a clear trend towards reduction of myelin markers in the CB and BS of *Dars1* ^*M256L/−*^ mice, which may reflect potential myelination or oligodendrocyte defects in hindbrain areas of *Dars1* ^*M256L/−*^ mice.

**Fig. 6:**
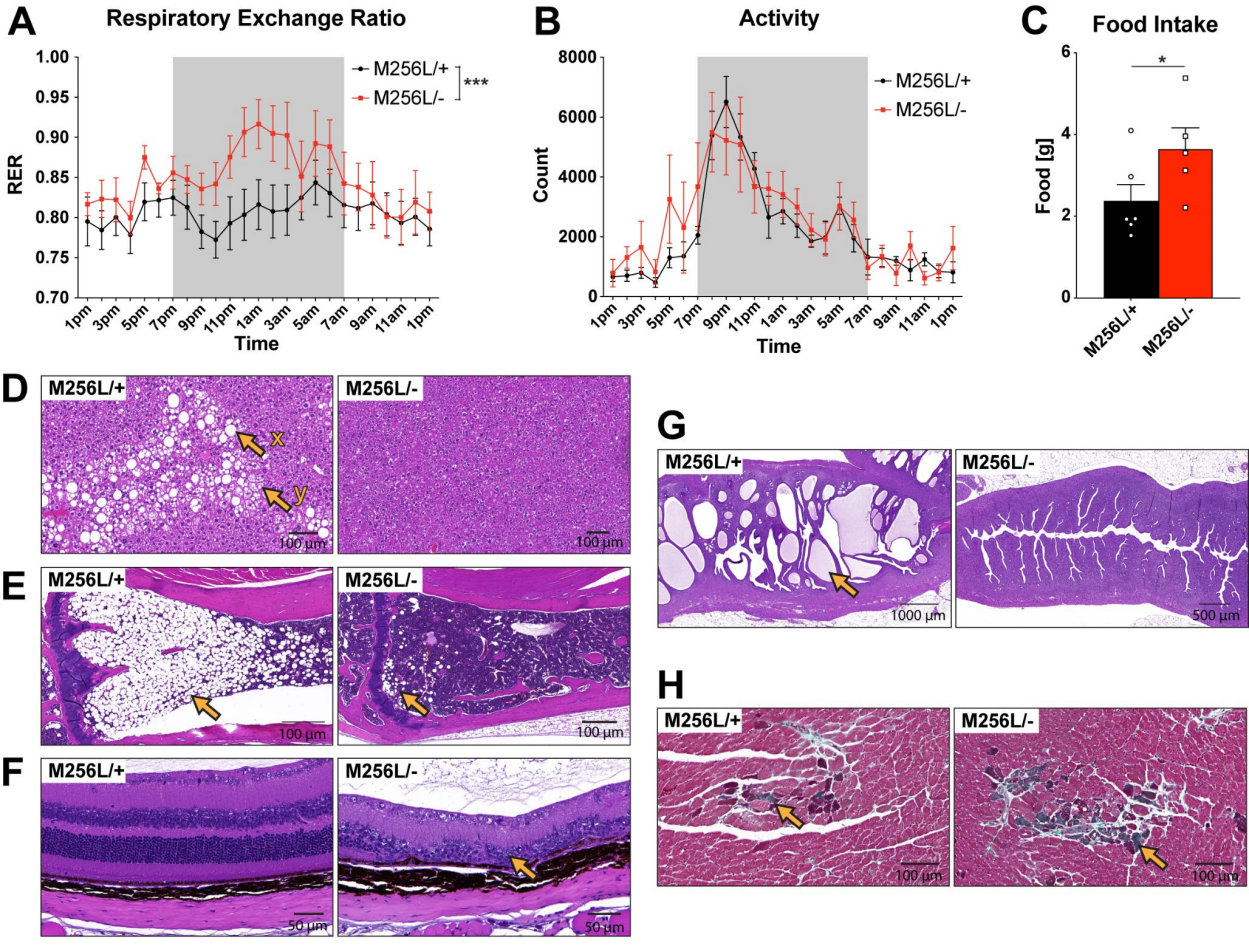
Respirometry analysis and histological assessment of peripheral organs of compound heterozygous *Dars1* ^*M256L/−*^ mice (M256L/-). (A-C) Simultaneous measurement of metabolic parameters in 10-month-old mice over a 24 h period employing CLAMS respirometry. (A) The respiratory exchange ratio (RER) was significantly elevated in M256L/- mice during the dark cycle (7pm – 7am) compared to M256L/+ controls (n = 5–7; mean ± SEM; ***p < 0.001; Two-way ANOVA). (B) Activity measured as the total number of times infrared beams were broken per hour is displayed over a 24 h period. Locomotion activity was unchanged between genotypes (n = 5–7). (C) Food intake of M256L/- mice was increased compared to M256L/+ controls (n = 5–6; mean ± SEM; (*p < 0.05; Student’s t-test). (D-G) Hematoxylin and Eosin (H&E) staining of peripheral organs of 11-month-old M256L/- and M256L/+ mice. (D) Reduced incidence and severity of macrovesicular (x) and microvesicular (y) steatosis (fatty change) in the liver of M256L/- mice. (E) Reduced hypocellularity (hematopoietic cells are replaced by adipocytes) of the bone marrow in the tibia of M256L/- mice (arrow). (F) Retinal degeneration (arrow) was observed in M256L/- mice but not in M256L/+ controls. (G) Reduced incidence and severity of uterine endometrial hyperplasia (arrow) in M256L/- mice. (H) Masson’s trichrome (MT) staining of the heart indicates increased incidence and severity of myocardial fibrosis (green stain; arrow) in 11-month-old M256L/- mice compared to M256L/+ controls

### Peripheral and metabolic changes in Dars1^M256L/-^ mice

EchoMRI body composition analysis (Fig. 2 F) revealed a reduction in fat mass of *Dars1* ^*M256L/−*^ mice compared to *Dars1* ^*M256L/+*^ controls, suggesting potential metabolic changes. To explore the underlying cause for the shift from fat to lean mass, we subjected *Dars1* ^*M256L/−*^ mice to respirometry analysis employing the Comprehensive Laboratory Animal Monitoring System (CLAMS), which allows for simultaneous measurement of metabolic parameters such as oxygen consumption, carbon dioxide production and metabolic exchange ratio, as well as food intake and activity levels. Mice were held individually in CLAMS cages for 24 h acclimatation, then measurements were taken over a 24 h period. The RER was significantly elevated in *Dars1* ^*M256L/−*^ mice during the dark cycle (7pm – 7am) compared to *Dars1* ^*M256L/+*^ controls (Fig. 6 A). The RER is the ratio between the carbon dioxide produced by the mouse and the oxygen consumed, which can be used as an indicator for the primary fuel source that is being metabolized, e.g., carbohydrates or fats. A mixed diet in healthy individuals normally results in a RER of 0.8. A value closer to 0.7 indicates lipid oxidation, while a RER closer to 1.0 implies that carbohydrates are primarily metabolized [18]. In *Dars1* ^*M256L/−*^ mice, the RER increased from 0.8 to 0.9 during the dark cycle (active phase) indicating a shift to carbohydrates as predominant fuel source (Fig. 6 A). In contrast, the RER of *Dars1* ^*M256L/+*^ mice remained at around 0.8, implying a balance between carbohydrates and fats as fuel sources (Fig. 6 A). Activity levels increased for both genotypes during the dark cycle without significant differences (Fig. 6B). Food intake, on the other hand, was significantly increased in *Dars1* ^*M256L/−*^ mice compared to *Dars1* ^*M256L/+*^ controls (Fig. 6 C). Histological analysis of peripheral organs of 11-month-old mice revealed a reduced incidence and severity of macrovesicular and microvesicular steatosis in the liver of *Dars1* ^*M256L/−*^ mice compared to *Dars1* ^*M256L/+*^ controls (Fig. 6D). Liver steatosis, commonly known as fatty liver disease, is an abnormal retention of lipids in the liver. Moreover, *Dars1* ^*M256L/−*^ mice displayed significantly reduced hypocellularity of the bone marrow (Fig. 6E). Hypocellular bone marrow is an indicator that hematopoietic cells were replaced by adipocytes. In addition to microphthalmia and anophthalmia present in 22% of the *Dars1* ^*M256L/−*^ mice (Fig. 2G), we observed retinal degeneration in *Dars1* ^*M256L/−*^ mice, signifying particular susceptibility of the eyes to AspRS impairment (Fig. 6 F). In female *Dars1* ^*M256L/−*^ mice, we also found reduced incidence and severity of uterine endometrial hyperplasia (Fig. 6G). Finally, Masson’s trichrome staining of the heart revealed increased incidence and severity of myocardial fibrosis, an excess deposition of collagen in the cardiac muscle of aged *Dars1* ^*M256L/−*^ mice compared to *Dars1* ^*M256L/+*^ controls (Fig. 6 H). Taken together, peripheral changes in *Dars1* ^*M256L/−*^ mice include considerable differences in fat metabolism and storage, abnormal eye development, as well as heart abnormalities.

## Discussion

Accurate animal models of the leukodystrophy HBSL have been challenging to establish in the past, as *Dars1* mutations have either resulted in severe developmental deficits and premature death or failed to trigger HBSL pathology altogether. In a first attempt at modelling HBSL, we characterized *Dars1*-knockout mice. The lack of redundancy of the AspRS enzyme rendered homozygous *Dars1*-null mice unviable, while heterozygous *Dars1*-null carriers lacked HBSL symptoms [8]. Results from this study suggested that successful modelling of HBSL requires the generation of different, disease-causing genocopies in mice to replicate the full disease spectrum. Recently, we described mice carrying the hypomorphic *Dars1* ^*D367Y*^ mutation *in trans* to the *Dars1*-null allele. Hypomorphs are mutations that reduce gene function without completely abolishing it. Consequently, the effect of a hypomorph is enhanced *in trans* to a deletion allele. These mice presented with early developmental delay, hydrocephalus, hypomyelination, white matter vacuolization and late onset motor impairment and thus represent a model of severe HBSL forms [9]. Here, we introduced another HBSL-causing missense mutation into the mouse genome using CRISPR/Cas9 gene editing. The *Dars1* ^*M256L*^ mutation has been described as the genetic cause of HBSL when homozygously present in patients [1]. In contrast to humans, mice carrying the *Dars1* ^*M256L*^ mutation homozygously did not develop any HBSL signs. This is in line with observation made with homozygous *Dars1* ^*D367Y*^ mice [9]. To trigger HBSL-like disease in *Dars1* ^*M256L*^ mice, we bred this mouse strain with *Dars1*-null carriers, as performed before for *Dars1* ^*D367Y*^ mice. The *Dars1* ^*M256L*^ mutation is also a hypomorph and the phenotype of mice carrying this mutation *in trans* to a *Dars1*-null mutation (*Dars1* ^*M256L/−*^) was significantly worse compared to homozygous *Dars1* ^*M256L*^ mice. Compound heterozygous *Dars1* ^*M256L/−*^ mice displayed increased embryonic lethality, severe developmental delay, reduced body weight and size, hydrocephalus, microphthalmia or anophthalmia, and vacuolization of the spinal cord white matter. Similar deficits have been described for the *Dars1* ^*D367Y/−*^ mouse strain [9]. Additionally, the *Dars1* ^*M256L/−*^ mutation genotype seems to have profound effects on energy metabolism evident through reduced body fat, increased RER as well as reduced liver steatosis and reduced hypocellularity of the bone marrow. In summary, while not recapitulating the full spectrum of the human HBSL disease, *Dars1* ^*M256L/−*^ mice can be leveraged to examine peripheral changes associated with *Dars1* deficiency.

Protein synthesis is an essential housekeeping function of all cells and mutations in components of the protein synthesis machinery are not tolerated. In the clinical context, mutations in AaRS-encoding genes primarily manifest in severe neurological diseases such as encephalopathies, neuropathies, cerebellar ataxia and leukodystrophies [4]. A comparable prevalence of nervous system defects was observed in *Dars1*^*D367Y/−*^ mice [9]. Likewise, aged *Dars1* ^*M256L/−*^ mice showed vacuolization of the lateral white matter of the thoracic region of the spinal cord accompanied by a reduction of myelin markers in the hindbrain. Vacuolization is a common feature of many demyelinating pathologies and is a hallmark of the leukodystrophies Vanishing white matter disease and Canavan disease [19]. In most cases, white matter vacuolization occurs in the context of demyelination and is then termed spongiform degeneration [19]. Despite the reduction of myelin proteins in the hindbrain, we did not observe overt signs of demyelination in the brain or spinal cord of *Dars1* ^*M256L/−*^ mice.

A common deficit of *Dars1* ^*D367Y/−*^ [9] and *Dars1* ^*M256L/−*^ (this study) mice was the severe developmental delay, which manifested in high embryonic mortality, reduced body size and weight, hydrocephalus and eye abnormalities such as microphthalmia, anophthalmia and retinal degeneration. Despite the devastating impact on early development, both mutation phenotypes were relatively innocuous after weaning (~ 3 weeks onwards). A growing organism has an increased demand for protein synthesis and during development, protein synthesis needs to be tightly regulated across cell types to establish individual cell identities [20]. Disturbances to this process during the early stages of development appear to be particularly impactful and result in the severe developmental defects observed in both genotypes. Once both mouse strains reached adulthood, the remaining AspRS aminoacylation activity appeared to be sufficient to maintain normal tissue homeostasis until the effects of impaired protein synthesis become apparent again in aged mice, resulting in CNS abnormalities. The late onset deficits can be explained by an abnormal accumulation of unfolded proteins in the endoplasmic reticulum over the lifetime of the mouse due to impaired AspRS aminoacylation activity. Under normal circumstance, the unfolded protein response (UPR) protects cells from endoplasmic reticulum stress, however, if the accumulation of misfolded proteins persists, apoptosis is triggered [21–23]. Failure of the UPR to cope with prolonged endoplasmic reticulum stress is involved in the pathophysiology of many white matter diseases including Charcot-Marie-Tooth disease, Pelizaeus-Merzbacher disease, Vanishing White Matter disease and multiple sclerosis [22] and was proposed as the potential underlying disease mechanism for HBSL [3].

Hydrocephalus, the pathological build-up of excess fluid in the brain, was another common feature developed by *Dars1* ^*D367Y/−*^ [9] and *Dars1* ^*M256L/−*^ mice during the first month of life. In humans, hydrocephalus has so far only been observed in one HBSL patient who was treated with ventriculo-peritoneal shunting to relieve the pressure from the brain [24]. Also present in both, *Dars1* ^*M256L/−*^ and *Dars1* ^*D367Y/−*^ mice, were severe eye abnormalities including microphthalmia, anophthalmia and retinal degeneration. Microphthalmia is a developmental eye disorder arising before birth and resulting in abnormally small eyeballs, while anophthalmia describes the complete absence of an eye [25]. There is a phenotypic continuum between the two conditions with anophthalmia representing the most severe form [25]. The fact that developmental defects of the eyes occurred in *Dars1* ^*M256L/−*^ as well as in *Dars1* ^*D367Y/−*^ mice implies that they are a direct consequence of AspRS dysfunction.

Behavioral deficits arising from the *Dars1* ^*M256L*^ mutation included reduced ASR and PPI as well as a reduction of the time spent in the inner compartment of an open field apparatus. The ASR involves a simple neuronal circuit of the lower brainstem including neurons of the caudal pontine reticular nucleus [16]. PPI of the ASR provides a measurement for sensorimotor gating mechanisms and involves a complex interplay of brain regions [16]. While the ASR was significantly lowered in homozygous *Dars1* ^*M256L*^ mice compared to wildtype controls, PPI was impaired in compound heterozygous *Dars1* ^*M256L/−*^ mice. The same observation was previously made for the *Dars1* ^*D367Y*^ line. Homozygously, the *Dars1* ^*D367Y*^ mutation resulted in a reduction of the ASR and when *in trans* to the null allele (*Dars1* ^*D367Y/−*^), reduced PPI was observed [9]. Consistently, heterozygous *Dars1*-null mice also exhibited reduced PPI of the ASR [8]. Taken together, *Dars1* missense mutations appear to result in reduced ASR, whereas the *Dars1*-null mutation leads to impaired PPI. A reduced PPI has been described for a variety of neurological disorders including schizophrenia and Huntington’s disease and appears to be also a feature of HBSL [9, 26]. The finding that *Dars1* ^*M256L/−*^ mice spent less time in the inner compartment indicates increased anxiety compared to *Dars1* ^*M256L/+*^ littermates, a result not previously reported in other rodent HBSL models.

Body composition analysis revealed significantly less body fat in *Dars1* ^*M256L/−*^ mice. Additionally, the RER was elevated in *Dars1* ^*M256L/−*^ mice during the dark cycle, which corresponds to the active phase of rodents. The RER is an indicator of the primary fuel source that is being used in metabolism and a high RER indicates carbohydrates as the predominant fuel source, whereas a low RER suggests lipid oxidation [18]. Furthermore, food intake of *Dars1* ^*M256L/−*^ mice was increased. Taken together, body fat, RER and food intake measurements were coherent and suggest that *Dars1* ^*M256L/−*^ mice have lowered fat storage, rely more on carbohydrates as a fuel source and consequently require increased food intake. Histological analysis of peripheral organs confirmed reduced fat deposition in *Dars1* ^*M256L/−*^ mice. Overall, based on metabolism and body composition data, *Dars1* ^*M256L/−*^ exhibited considerable vigor and have the appearance of younger mice.

In conclusion, despite being less severely affected, mice harboring the *Dars1* ^*M256L*^ mutation displayed a similar pattern of developmental and CNS deficits as previously observed in *Dars1* ^*D367Y*^ mice. Aside from a significant ASR reduction, homozygous *Dars1* ^*M256L*^ mice were unaffected and did not recapitulate the disease phenotype observed in homozygous HBSL patients. Compound heterozygous *Dars1* ^*M256L/−*^ mice carrying the hypomorphic *Dars1* ^*M256L*^ mutation *in trans* to a *Dars1*-null allele presented with severe developmental delay, spinal cord white matter vacuolization and reduction of myelin markers in the hindbrain. Intriguingly, we observed significant changes in peripheral organs possibly as a result of altered energy metabolism. The body composition of *Dars1* ^*M256L/−*^ mice differed compared to controls, with reduced fat mass, elevated RER and increased food intake. The genetic mouse model described in this study, although not completely recapitulating the clinical picture of HBSL, will help to unravel the underlying pathophysiology and provides new insight into the contribution of peripheral factors to the complex disease presentation. The peripheral signs resulting from AspRS deficiency could potentially be leveraged to establish non-invasive tools for differential HBSL diagnosis to support early detection and better patient outcomes. Together with the previously described rodent HBSL models, the new transgenics will be instrumental for testing of novel therapies across the entire disease spectrum, including *DARS1* gene therapy and nutraceutical aspartate supplementation [27]. The seemingly irreplaceable function of AspRS during early development indicates that a timely diagnosis, paired with supportive therapies, are essential to ensure progression beyond critical, juvenile stages to warrant a better outcome for HBSL patients.

## Data Availability

All data generated or analyzed during this study are included in this published article and will be made available on reasonable request.

## Abbreviations

HBSL: Hypomyelination with brainstem and spinal cord involvement and leg spasticity
AaRS: Aminoacyl-tRNA synthetase
AspRS: Aspartyl-tRNA synthetase
ARS: Acoustic startle response
PPI: Pre-pulse inhibition
RER: Respiratory exchange ratio
