# Correction to: Developmental delay and late onset HBSL pathology in hypomorphic *Dars1*^M256L^ mice

**DOI:** 10.1007/s11064-022-03602-3

**Published:** 2022-04-22

**Authors:** Matthias Klugmann, Elizabeth Kalotay, Fabien Delerue, Lars M. Ittner, Andre Bongers, Josephine Yu, Margaret J. Morris, Gary D. Housley, Dominik Fröhlich

**Affiliations:** 1 Translational Neuroscience Facility, Department of Physiology, School of Medical Sciences, University of New South Wales, 2052 Sydney, NSW Australia; 2 Dementia Research Centre, Department of Biomedical Sciences, Faculty of Medicine, Health and Human Sciences, Macquarie University, 2109 Sydney, NSW Australia; 3 Biomedical Resources Imaging Laboratory, University of New South Wales, 2052 Sydney, NSW Australia; 4 Department of Pharmacology, School of Medical Sciences, University of New South Wales, 2052 Sydney, NSW Australia

## Correction to: Neurochemical Research 10.1007/s11064-022-03582-4

In the original version of this article, unfortunately the figures and its captions were wrongly displayed. The Figs. 1, 2, 3, 4, 5, and 6 and its correct captions are given below.

Additionally, all in-text figure references were published incorrectly. Where Fig. 1 is referenced in the original text, it should read Fig. 4. Where Fig. 2 is referenced in the original text, it should read Fig. 6. Where Fig. 3 is referenced in the original text, it should read Fig. 1. Where Fig. 4 is referenced in the original text, it should read Fig. 2. Where Fig. 5 is referenced in the original text, it should read Fig. 3. Where Fig. 6 is referenced in the text original, it should read Fig. 5.

**Fig. 1:**
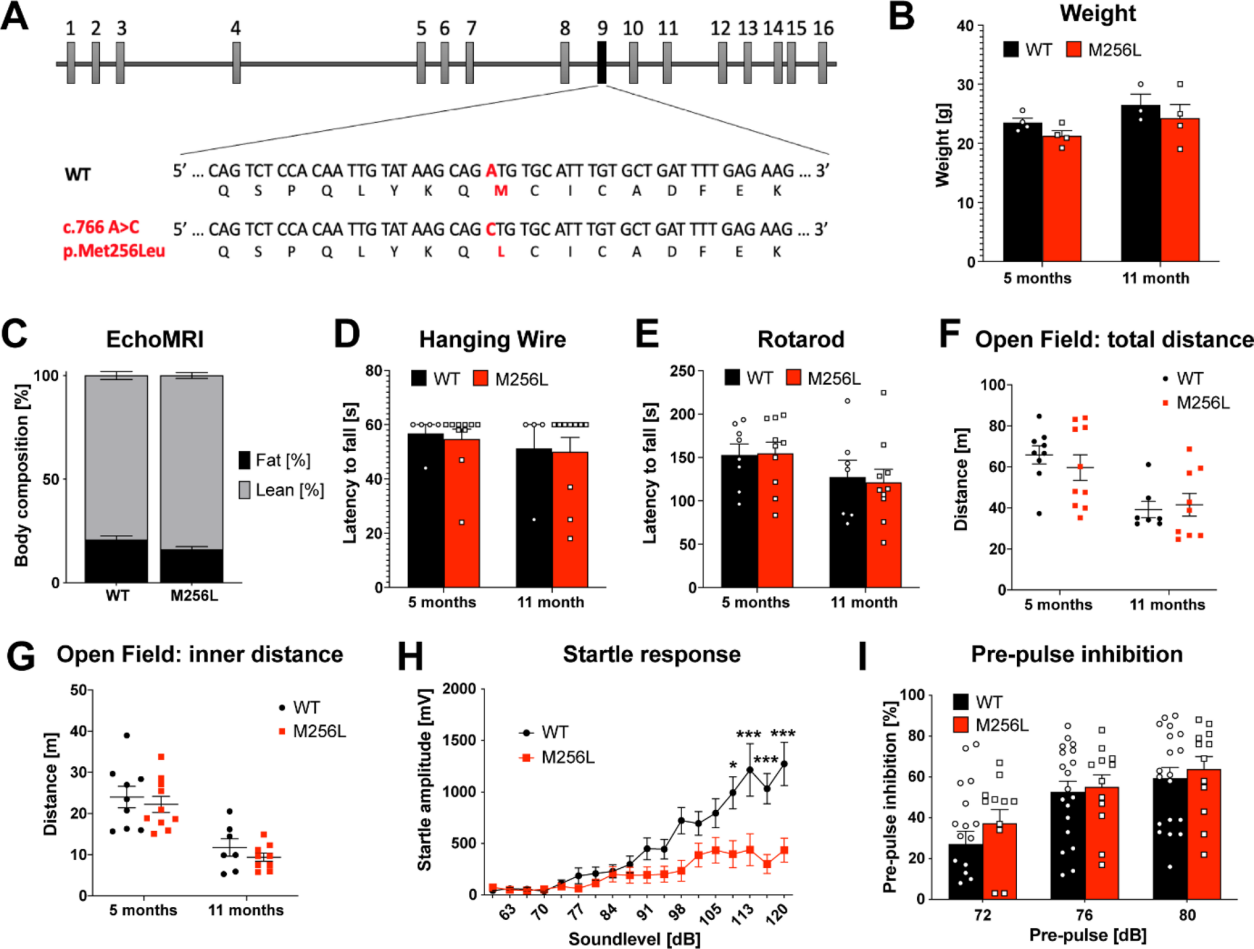
Phenotype of homozygous *Dars1*^*M256L/M256L*^ mice (M256L). **A** Illustration of the genomic location of the *Dars1* c.766 A>C; p.Met256Leu mutation on exon 9. **B** Body weight of M256L female mice compared to WT controls at 5 and 11 months of age (n = 3–4). **C** Body composition analysis using EchoMRI showed no significant differences between homozygous M256L and WT mice (n = 7–10). The hanging wire (**D**) and the rotarod test (**E**) revealed normal muscle strength and motor coordination of M256L mice compared to WT controls (n = 4–10). Total distance (**F**) and distance travelled in the inner compartment (**G**) of an open field test apparatus were comparable between M256L and WT mice (n = 7–10). **H** Five-month-old homozygous M256L mice exposed to 40 ms sound stimuli with increasing intensities (60–120 dB SPL) displayed reduced acoustic startle response compared to controls (n = 10–19). **I** No differences in pre-pulse inhibition (120 dB SPL startle pulse preceded by 72, 76, or 80 dB SPL pre-pulses) were observed in M256L mice compared to WT controls (n = 12–19). Data represent mean ± SEM (*p  < 0.05, ***p  < 0.001; Two-way ANOVA)

**Fig. 2:**
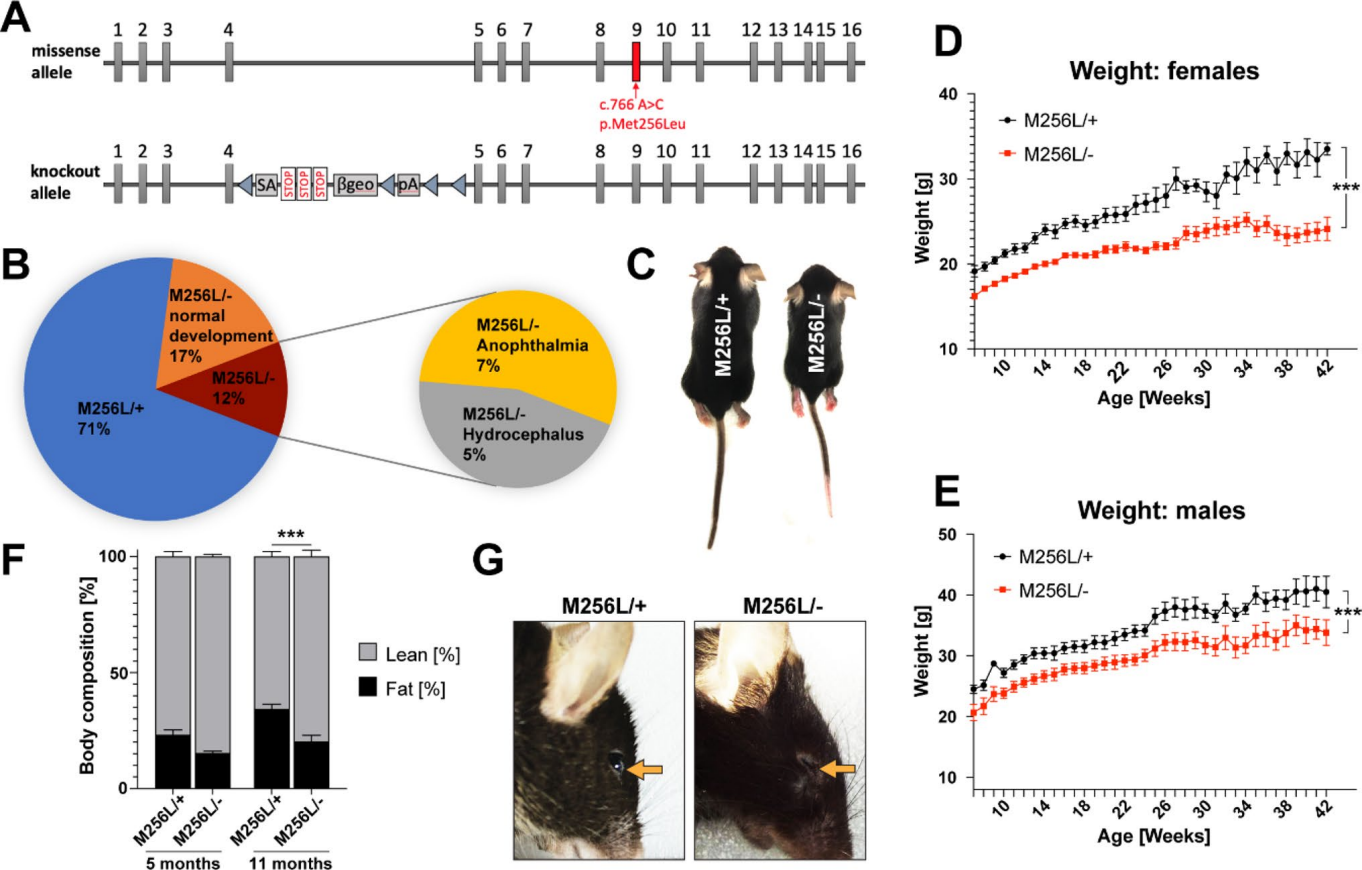
Developmental deficits of compound heterozygous *Dars1*^*M256L/−*^ mice (M256L/−). **A** Schematic depicting the set of *Dars1* alleles present in compound heterozygous M256L/− mice. Allele 1 contains the c.766 A>C; p.Met256Leu missense mutation while allele 2 is the *Dars1*-null allele described in Fröhlich et al., 2017. **B** Genotype and phenotype distribution in the F1 offspring of homozygous M256L and heterozygous *Dars1*-null mice (n = 94; 19 litters). **C** M256L/− mice show reduced body size compared to age- and sex-matched M256L/+ littermates. Weight of M256L/+ and M256L/− females (**D**; n = 5–11) and males (**E**; n = 3–11). **F** EchoMRI body composition shows a shift from fat to lean mass in M256L/− mice at 5 months with a significant reduction in body fat in M256L/− mice compared to M256L/+ littermates at 11 months (n = 7–9). **G** 22% of the viable M256L/− mice (7% of the total F1 mice) showed various degrees of anophthalmia or microphthalmia (arrow). Data represent mean ± SEM (***p < 0.001; Two-way ANOVA)

**Fig. 3:**
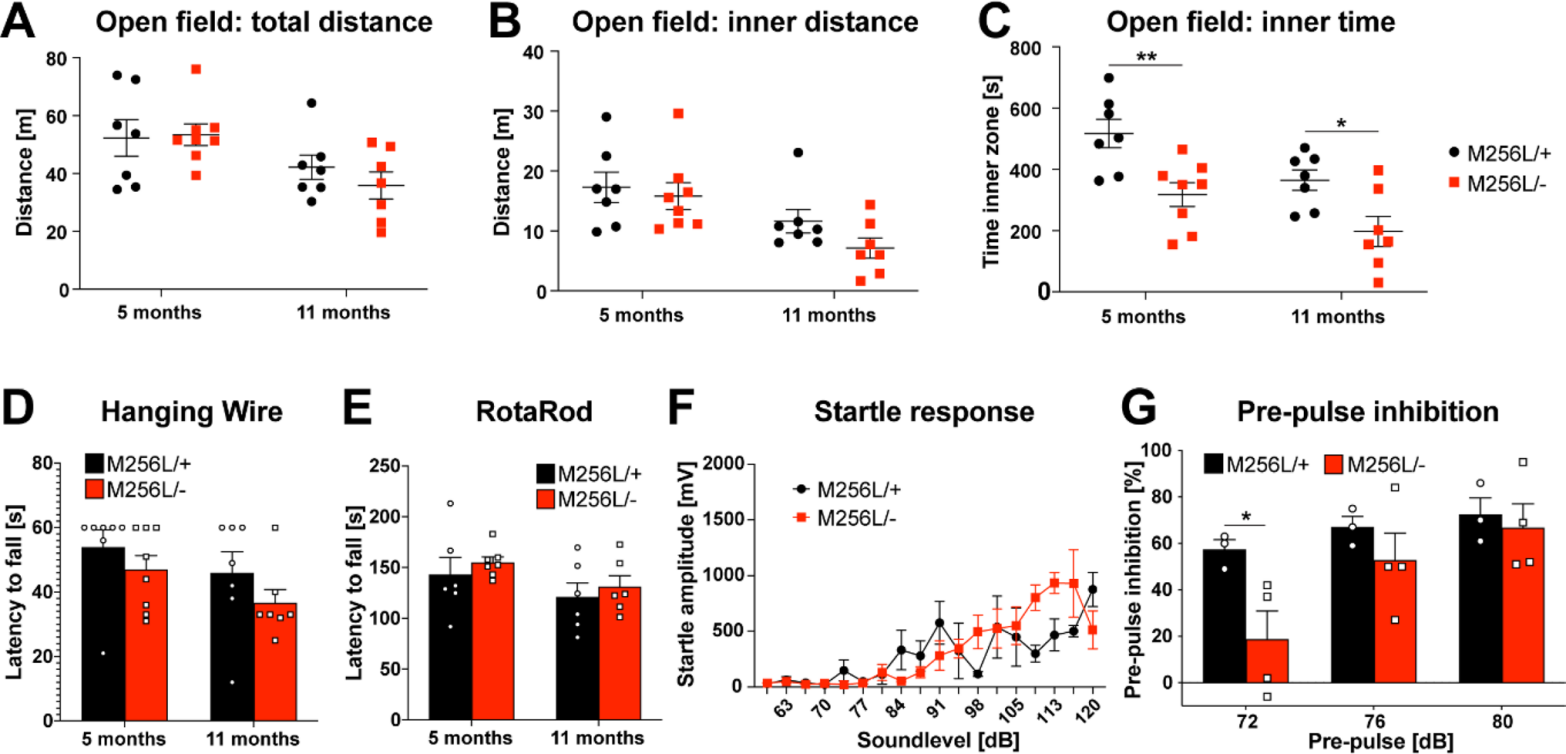
Behavioral assessment of compound heterozygous *Dars1*^*M256L/−*^ mice (M256L/−). **A**–**C** Total distance, distance in the inner compartment and time spent in the inner compartment of an open field test apparatus were assessed (n = 7). M256L/− mice spent significantly less time in the inner (open) compartment compared to M256L/+ controls (**C**). **D** and **E** Muscle strength and motor coordination assessed by the hanging wire and rotarod tests were unaffected in M256L/− mice compared to M256L/+ controls (n = 7). **F** Acoustic startle responses following sound stimuli with increasing intensities (60–120 dB SPL, 40 ms) were unaltered between M256L/– and M256L/+ mice (n = 4). **G** Pre-pulse inhibition (PPI) through 72, 76 or 80 dB SPL pre-pulses played 100 ms before the 120 dB SPL startle pulse was measured. A significant reduction of PPI was observed in M256L/− mice following the 72 dB SPL pre-pulse (n = 3–4). Data represent mean ± SEM (*p  < 0.05, ** *p *< 0.01; Two-way ANOVA)

**Fig. 4:**
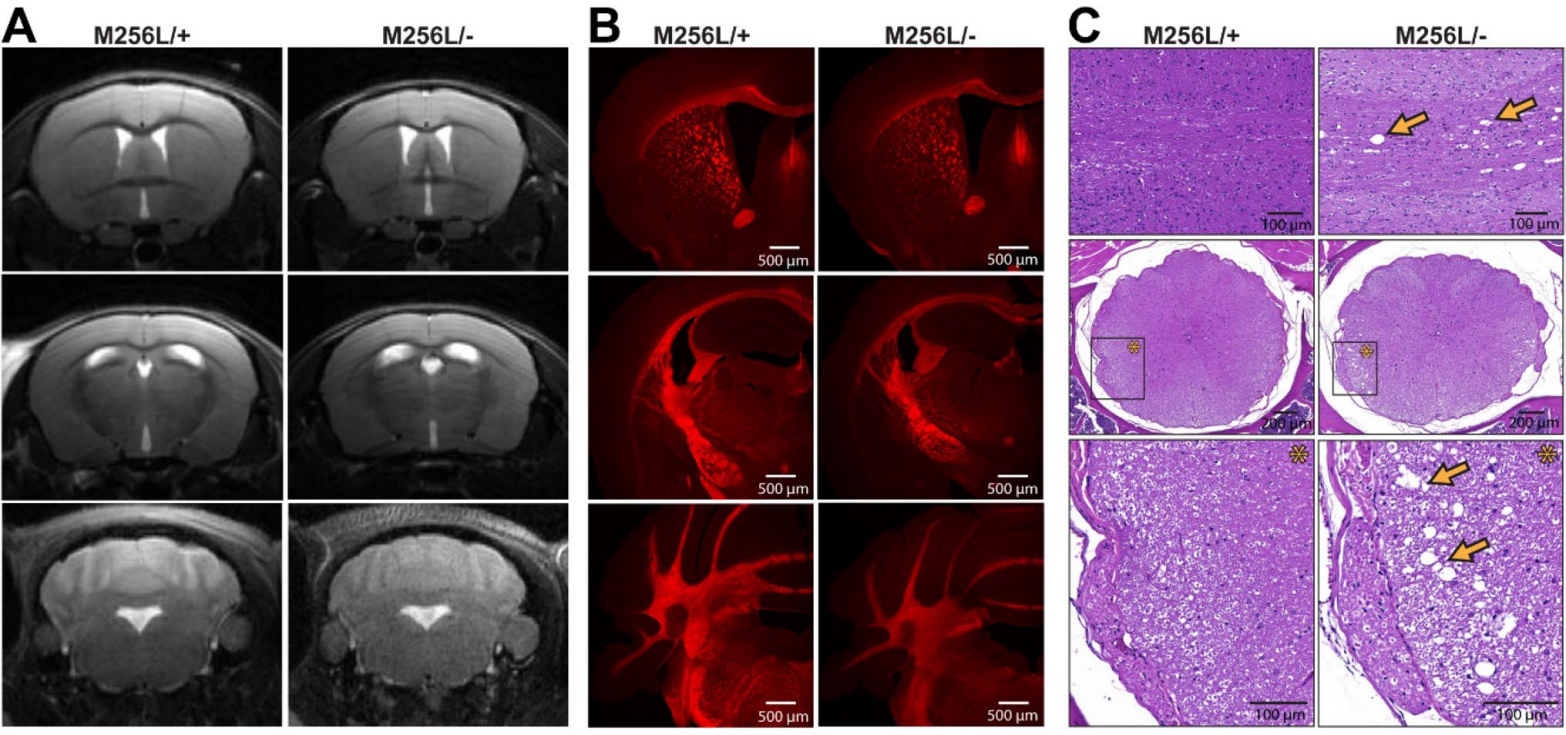
CNS morphology and myelination of compound heterozygous *Dars1*^*M256L/−*^ mice (M256L/−). **A** Brain MRI did not show overt morphological or myelination abnormalities of M256L/− mice compared to M256L/+ littermates. **B** FluoroMyelin Red staining of coronal brain sections revealed no differences in myelination of M256L/− and M256L/+ mice. **C** Hematoxylin and Eosin (H&E) staining of longitudinal sections (top) and thoracic cross sections (middle and bottom) of the spinal cord showed vacuolization of the lateral white matter (arrows) in 11-month-old M256L/− mice but not in M256L/+ controls

**Fig. 5:**
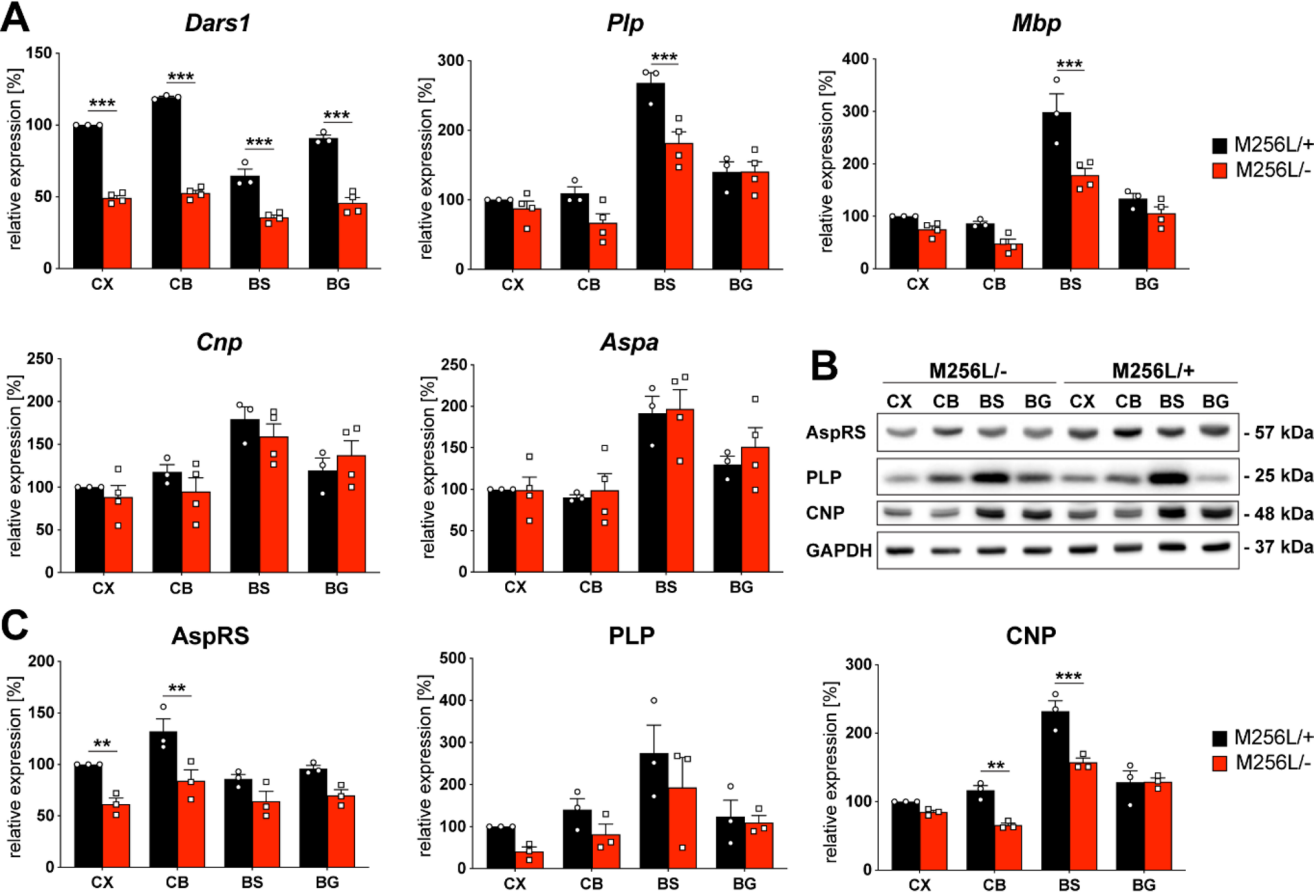
mRNA and protein levels of *Dars1*/AspRS and the major myelin proteins in *Dars1*^*M256L/−*^ mice (M256L/−). **A** mRNA expression levels assessed by qPCR in different brain regions (CX, cortex; CB, cerebellum; BS, brainstem; BG, basal ganglia) of 11-months old M256L/− mice compared to M256L/+ controls. Expression was normalized to the housekeeper Gusb (n = 3–4). **B** Representative Western-blot indicating expression of AspRS, the myelin proteins PLP and CNP, and the housekeeping protein GAPDH in the brain of M256L/− and M256L/+ mice. **C** Densitometric quantification of AspRS, PLP and CNP protein levels normalized to the housekeeper GAPDH in different brain regions of 11-month-old M256L/− mice compared to M256L/+ controls (n = 3). Data represent mean ± SEM (** *p * < 0.01, ***p  < 0.001; Two-way ANOVA).

**Fig. 6:**
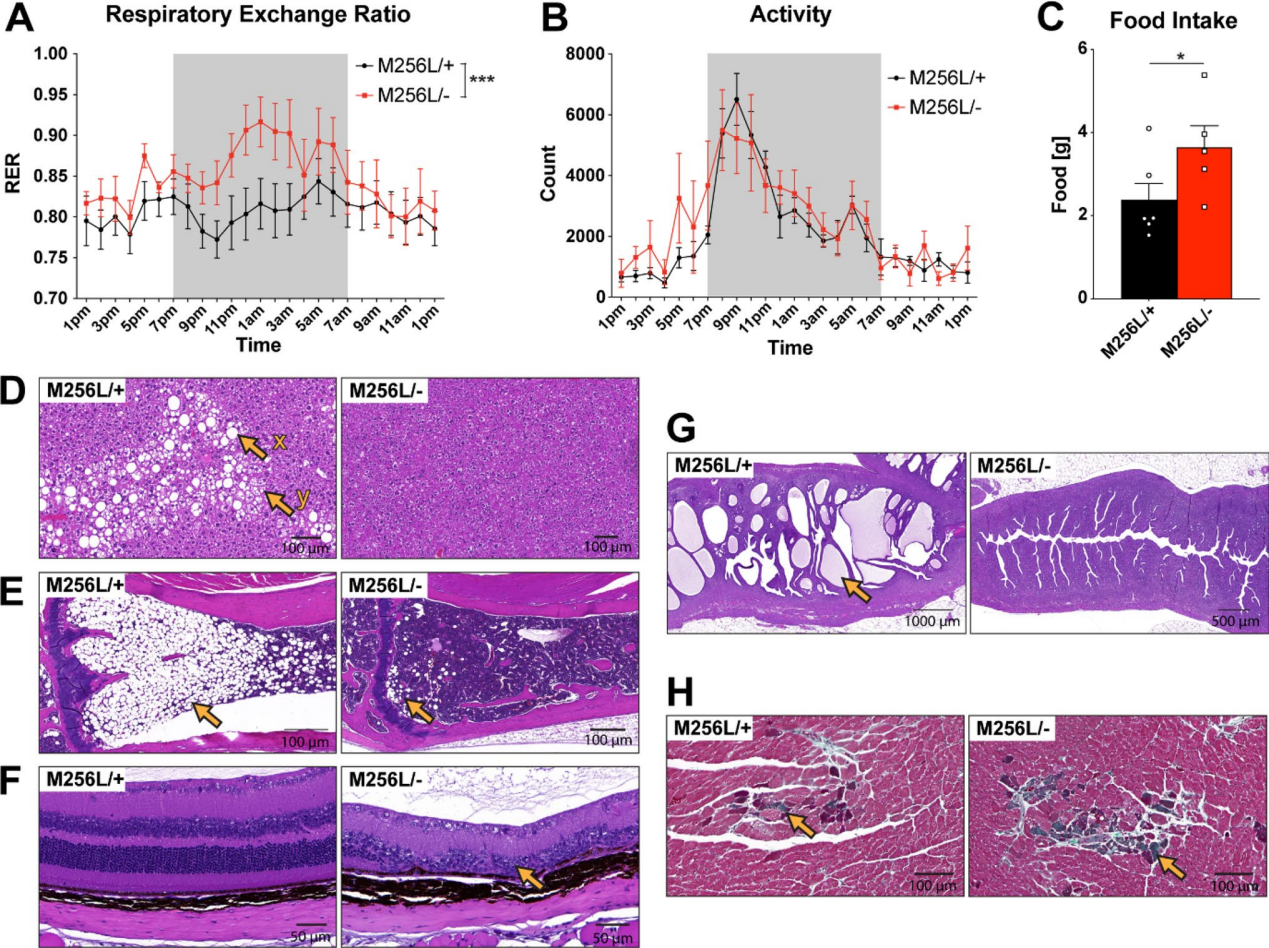
Respirometry analysis and histological assessment of peripheral organs of compound heterozygous *Dars1*^*M256L/−*^ mice (M256L/−). **A**–**C** Simultaneous measurement of metabolic parameters in 10-month-old mice over a 24 h period employing CLAMS respirometry. **A** The respiratory exchange ratio (RER) was significantly elevated in M256L/− mice during the dark cycle (7 pm–7am) compared to M256L/+ controls (n = 5–7; mean ± SEM; ***p  <  0.001; Two-way ANOVA). **B** Activity measured as the total number of times infrared beams were broken per hour is displayed over a 24 h period. Locomotion activity was unchanged between genotypes (n = 5–7). **C** Food intake of M256L/− mice was increased compared to M256L/+ controls (n = 5–6; mean ± SEM; (*p  <  0.05; Student’s t-test). **D**–**G** Hematoxylin and Eosin (H&E) staining of peripheral organs of 11-month-old M256L/− and M256L/+ mice. **D** Reduced incidence and severity of macrovesicular (x) and microvesicular (y) steatosis (fatty change) in the liver of M256L/− mice. **E** Reduced hypocellularity (hematopoietic cells are replaced by adipocytes) of the bone marrow in the tibia of M256L/− mice (arrow). **F** Retinal degeneration (arrow) was observed in M256L/− mice but not in M256L/+ controls. **G** Reduced incidence and severity of uterine endometrial hyperplasia (arrow) in M256L/− mice. **H** Masson’s trichrome (MT) staining of the heart indicates increased incidence and severity of myocardial fibrosis (green stain; arrow) in 11-month-old M256L

The original article has been corrected.

